# Fatal Case of Pulmonary Invasive Aspergillus after Heart Transplant with a Rapidly Progressive Course

**Published:** 2018-08-01

**Authors:** B. Shakerian, N. Razavi, M. H. Mandegar

**Affiliations:** 1 Department of Cardiovascular Surgery, Tehran University of Medical Sciences, Tehran, Iran; 2 Department of Genetics, Shahrekord Azad University, Shahrekord, Iran

**Keywords:** Aspergillus, Heart transplant, Fungal infection

## Abstract

The incidence of invasive fungal infections is lower than that of bacterial infections in heart transplant recipients. However, they are always life-threatening. Clinical manifestations may range from asymptomatic colonization to disseminated infection. This complication is responsible for significant morbidity and mortality, particularly in heart transplant recipients. Herein, we present on a cardiac transplant recipient who presented with invasive pulmonary aspergillosis quickly leading to death, in spite of early diagnosis and aggressive therapy. It just took 10 hours from the diagnosis to death. In other reports, this period was at least 12 days.

## INTRODUCTION

*Aspergillus fumigatus* is the most common invasive fungal infection affecting heart transplant recipients. The overall incidence of the invasive aspergillosis has decreased over the last few years. It is usually a relatively early complication post-cardiac transplantation. Although invasive aspergillosis is a serious disease in heart transplantation, little is known about its natural history.

## CASE PRESENTATION

**Figure 1:**
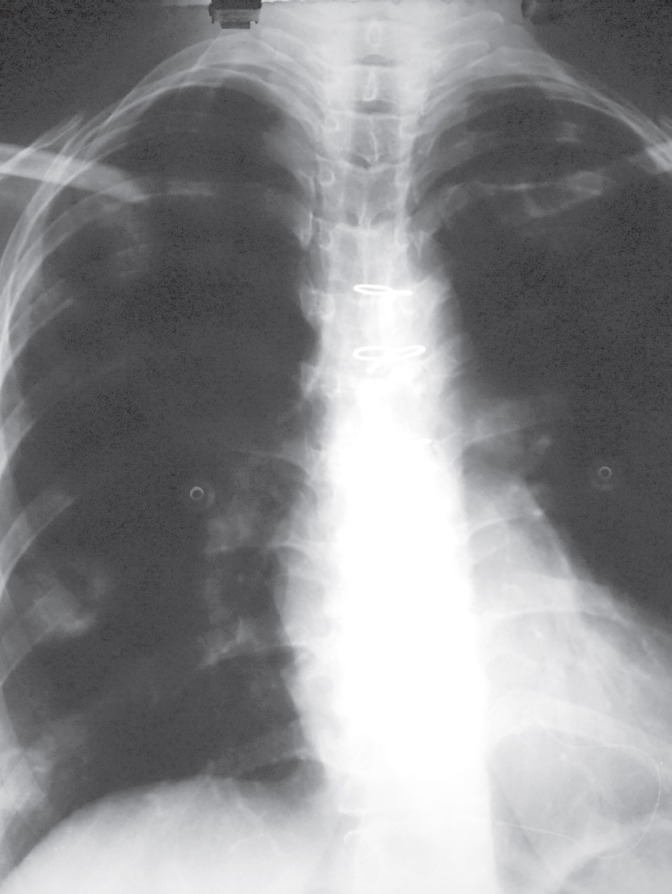
Normal chest x-ray at the time of initial presentation

**Figure 2:**
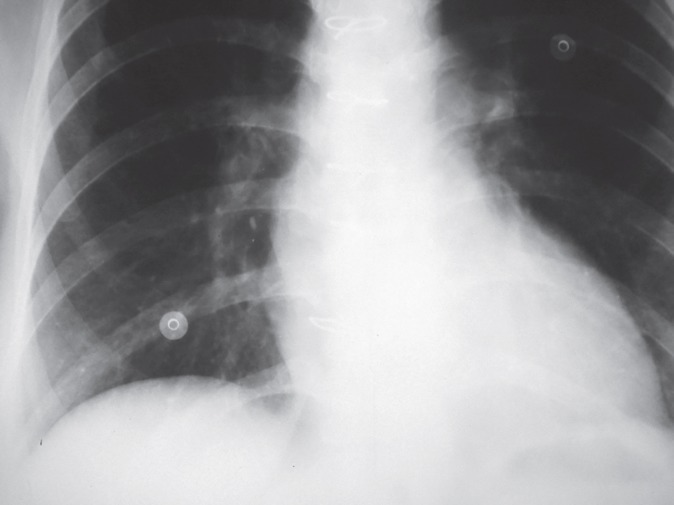
Chest x-ray showing infiltration in the right-lower and middle lobes

**Figure 3:**
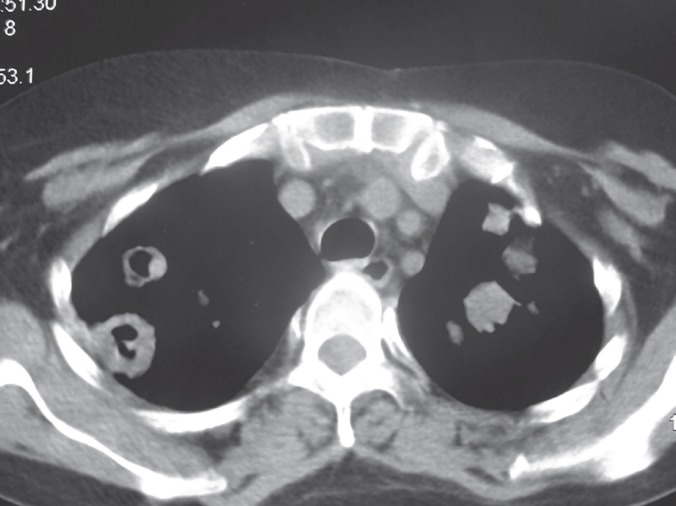
Computed tomography showed multiple consolidation with halo sign of both lungs

A 32-year-old woman, a known case of idiopathic dilated cardiomyopathy with progressive heart failure presented with New York Heart Association class 3–4 heart failure and underwent orthotopic heart transplantation (HTx). Her immunosuppressant regimen included anti-thymocyte globulin, prednisolone, mycophenolate mofetil, and tacrolimus. Her post-operative course was uneventful and she was discharged in excellent condition on 12^th^ post-operative day. Four months later, she presented with dry cough of two days duration. The patient denied any fever, chills, hemoptysis, chest pain, myalgia, orthopnea, and palpitation. Vital signs included a blood pressure of 120/70 mm Hg, oral temperature of 37.2 °C, heart rate of 78 beats/min, respiratory rate of 18 breath/min, and O_2_ saturation of 94% at room temperature. In physical examination, normal heart sounds and clear breathing sounds were noted. Laboratory blood tests showed total white blood cell count of 8200/mm^3^, 65% neutrophils, 28% lymphocyte, 4% monocytes and 3% eosinophils. Cytomegalovirus (CMV) was negative. Renal and liver function tests were within normal range. Chest x-ray was normal (Fig 1). An echocardiogram showed an ejection fraction of 50%, which was unchanged from the previous results. After six hours, she developed dyspnea and low-grade fever. Repeated chest x-ray revealed a new infiltration involving the right-middle and lower lobes (Fig 2). On suspicion of pneumonia, bronchoscopy and bronchoalveolar lavage were performed. Blood, urine, and stool culture were obtained and empiric broad-spectrum antibiotic therapy was initiated. Computed tomography of chest revealed bilateral multiple well-defined consolidations with halo sign (Fig 3). At this stage, clinical diagnosis of invasive pulmonary aspergillosis was made and antifungal therapy was initiated with voriconazole and amphotericin B. Prednisolone was discontinued. The immunosuppressant doses were lowered. Serum Aspergillus galactomannan antigen assay was requested. The condition of the patient worsened; she developed sudden-onset respiratory failure, necessitating endotracheal intubation and mechanical ventilation. Her hemodynamic became unstable, requiring vasopressor support. After two more hours the patient worsened clinically and subsequently succumbed to cardiorespiratory arrest. Blood, urine, and stool cultures showed no microbial growth after four days of incubation. The test for CMV antigenemia was negative. Multiple bronchoalveolar lavage cultures isolated *A. fumigatus*.

## DISCUSSION

HTx is the treatment of choice for patients with congestive heart failure. HTx recipients are at high risk of infections. The most common infections include pulmonary infections. Aspergillus is naturally presented in water, dust, soil, and building materials. Aspergillus infection is the most common cause of all invasive fungal diseases, accounting for 69.8% of total infections post-HTx [1]. *A. fumigatus* is the most common cause of invasive pulmonary aspergillosis. However, other species have also been reported. The incidence of invasive Aspergillosis in HTx recipients varies between 1% and 14% [2]. More than 75% of these infections occurred within the first three months after transplantation. This period coincidences with the maximum immunosuppression and intensive care unit stay. Cases with late-onset Aspergillosis (>3 months) after HTx carry a higher risk of mortality and dissemination than those with early-onset disease [3]. Risk factors for invasive Aspergillosis include reoperation, hemodialysis, CMV infection, diabetes mellitus, and heart-kidney transplantation. Recently, Rabin, *et al*, described that risk factors for invasive fungal infections consist of delayed chest closure and additional induction with OKT3, anti-thymocyte globulin, or daclizumab [4]. In our patient, anti-thymocyte globulin was the only facilitating factor for fungal infection. There is no document showing pre-transplantation isolation of Aspergillus or positive environmental culture. Symptoms of invasive pulmonary Aspergillus are nonspecific, but fever and cough are the most frequent clinical symptoms in HTx recipients. Other symptoms consist of pleuretic chest pain, hemoptysis, respiratory distress, and hemoptysis. Rapid diagnosis of fungal infections in HTx recipients is very important. Diagnosis is based on isolation of Aspergillus species from the respiratory tract secretions or biopsies with visualization of hyphae in tissues. Percutaneous or open lung biopsies are more accurate techniques compared to sputum or bronchoscopic specimens, with up to 100% sensitivity and specificity [5]. Serum galactomannan assay shows moderate accuracy for the diagnosis of invasive Aspergillosis in immunocompromised patients [6]. Computed tomography helps in diagnosis of invasive Aspergillosis. Typical signs of invasiveness are peribronchial halo sign and multiple nodules on computed tomography. Prompt antifungal therapy is the most important aspects in attaining a successful outcome. Voriconazole is the drug of choice for treatment of invasive Aspergillosis. Duration of treatment for invasive Aspergillosis is typically a minimum of 12 weeks. Antifungal prophylaxis is not recommended in HTx patients due to low incidence of the disease, high cost and drugs interactions. The mortality rate is 53%–78% [7]. In conclusion, invasive Aspergillosis can be catastrophic complication in HTx recipients and is associated with high rate of mortality. Invasive Aspergillosis should always be considered in differential diagnosis of pneumonia in HTx recipients.
